# Metabolic risk assessment in children and adolescents using the tri-ponderal mass index

**DOI:** 10.1038/s41598-022-13342-7

**Published:** 2022-06-16

**Authors:** Young-Jun Seo, Young Suk Shim, Hae Sang Lee, Jin Soon Hwang

**Affiliations:** 1 Department of Pediatrics, Hallym University Chuncheon Sacred Heart Hospital, 77 Sakju-ro, Chuncheon-si, Gangwon-do, 24253 Republic of Korea; 2 Department of Pediatrics, Ajou University Hospital, Ajou University School of Medicine, 164 World Cup-ro, Yeongtong-gu, Suwon, 16499 Republic of Korea

**Keywords:** Epidemiology, Paediatric research, Endocrine system and metabolic diseases

## Abstract

We assessed the risk of metabolic syndrome in children and adolescents who were classified using the tri-ponderal mass index (TMI) with data from the Korea National Health and Nutrition Examination Survey (KNHANES). Data from 10 to 18-year-old subjects that were overweight or obese (n = 1362) were extracted from the KNHANES 2007–2018. Weight classifications were determined by TMI and included overweight and Class I, Class II, and Class III obesity. The standard deviation scores (SDS) of weight, waist circumference, and body mass index (BMI) as well as cardiometabolic risk factors, including blood pressure, serum glucose levels, total cholesterol (T-C), triglycerides, HDL-c, and low-density lipoprotein cholesterol (LDL-c), worsened with the severity of obesity. Most risk factors showed a linear association with the severity increase, except for fasting glucose levels, T-C, and LDL-c. The prevalence of cardiometabolic risks also increased with the severity of obesity, which developed earlier in boys than in girls. The risk of metabolic syndrome significantly increased with the severity of obesity in both unadjusted and adjusted analyses. TMI reflected the severity of obesity and predicted the risk of metabolic syndrome and its components. Therefore, clinical applications of TMI could be a useful to identify the incidence of childhood obesity and metabolic syndromes.

## Introduction

Childhood obesity is currently a global health problem. The prevalence of obesity has nearly doubled over the past 20 years, although the exact number depends on the definition of obesity employed in each study and differed by region. According to the WHO, the global prevalence of obese male and female children aged 5–19 years dramatically increased from 0.7 and 0.9% in 1975 to 5.6% and 7.8% in 2016, respectively^1^. If obesity is defined as BMI ≥ 95th percentile for age and sex, or 25 ≥ kg/m^2^, the prevalence of pediatric obesity in Korea has also rapidly increased from 5.8% in 1998^2^ to 9.8% in 2017 (KNHANES 2017)^3^. Obesity in children and adolescents is known to lead to obesity in adulthood, which causes metabolic syndromes (a cluster of conditions including hypertension, hyperlipidemia, and diabetes, mellitus and increased the risk of premature death due to early atherosclerosis)^4^. Thus, preventing children and adolescents from becoming overweight is considered an important factor in preventing obesity-induced metabolic syndrome in adults^5^. Overweight and obese children and adolescents have been shown to be more likely to become obese adults^6^. Indeed, the presence of the cardiometabolic risk factors in childhood strongly predicted cardiovascular disease (CVD) in adulthood; a risk factor even stronger than of sex or a family history of CVD^7,8^. Moreover, it is generally accepted that childhood obesity not only leads to obesity in adulthood but is in itself a risk factor for cardiometabolic syndrome and CVD in adulthood^9^.

Classification of obesity provided a more precise assessment of the risks of morbidity and mortality and allows timely intervention. Several studies have attempted to classify obesity in children and adolescents according to the severity of adiposity. Srinivasan et al. reported that the incidence of adult metabolic abnormalities was positively correlated with childhood BMI quartile^10^. Similarly, Zhang et al. showed that if childhood obesity was classified by severity and type, severe abdominal obesity was positively associated with metabolic abnormalities^11^. Recently, a cross-sectional study based on data from the Korea National Health and Nutrition Examination Survey (KNHANES) 2007–2014 investigated metabolic risk factors according to the BMI of Korean adolescents; they reported that the severity of obesity (overweight, moderate, or severe) influenced metabolic risk factors^12^.

Several indirect methods have been used to estimate the adiposity of children and adolescents such as BMI, WC, the waist-to-hip ratio, and skinfold thickness. Among these indices, BMI is the most widely used to diagnose and categorize obesity; recent guidelines have also recommended using normative BMI percentiles^13^. However, in contrast to adults, bodyweight is not proportional to height squared in children and adolescents, which reduces the validity of BMI in diagnosing obesity at these ages^14,15^. Additionally, BMI z scores have been applied to problems that do not accurately reflect the degree of obesity in children; however, this dose not address the issue that body proportions (weight and height) and body adiposity are not proportional during this growth period and thus BMI should not be used to estimate adiposity.

To rectify these issues, recently, tri-ponderal mass index (TMI) has been recently proposed as a reasonable proxy for body adiposity. This index has several advantages, such as a lower misclassification rate, better reflection of body adiposity, and a more constant value of assessing body adiposity during the growth period than that of BMI z scores^16^. In a cross-sectional population-based study, we also recently reported that TMI was superior to BMI to discriminate metabolic risk factor^17^. Although TMI has been considered a more precise index for assessing childhood obesity, there are insufficient studies related to cardiovascular risk according to the degree of obesity. In the present study, we aimed to investigate the association between the severity of obesity and current cardiometabolic risk factors via a cross-sectional analysis of KNHANES data during 2007–2018. For achieve a more accurate analysis, we attempted to use a TMI-based classification to define the severity of obesity.

## Results

### Basic characteristics of the study participants

Among 7262 subjects who have data for anthropometrical and laboratory evaluation, the number of overweight or obese subjects for cardiovascular risk assessment was 1362 (729 boys, 633 girls). The distribution of adiposity classifications (overweight and Class I, Class II, and Class III obesity) was 12.6% (915/7262), 3.6% (259/7262), 1.6% (117/7262), and 1% (71/7262), respectively. The mean age, fasting glucose levels, T-C, TG and LDL-c, and the mean percent of individuals who consumed alcohol and had a household income < 2nd quartile did not differ between boys and girls. However, the SDSs of height, weight, WC, BMI, and TMI, and BP differed between boys and girls. There were sex differences in the HDL-c level, physical activity, and percent of individuals who smoked. The clinical characteristics of the participants are summarized in Table 1.

**Table 1:** Clinical characteristics of the study population according to sex (*n* = 1362).

	Boys (*n* = 729)	Girls (*n* = 633)	*P*
Age (years)	14.25 ± 2.47	14.31 ± 2.54	0.635
Height SDS	0.30 ± 1.06	0.07 ± 1.13	< 0.001
Weight SDS	1.79 ± 0.92	1.58 ± 0.90	< 0.001
WC SDS	1.35 ± 0.70	1.24 ± 0.90	0.012
BMI SDS	2.05 ± 0.75	1.87 ± 0.77	< 0.001
TMI SDS	1.50 ± 0.37	1.57 ± 0.43	0.002
SBP (mmHg)	114.68 ± 10.63	108.01 ± 10.05	< 0.001
DBP (mmHg)	68.68 ± 9.67	66.86 ± 8.47	< 0.001
Glucose (mg/dL)	92.32 ± 7.34	91.35 ± 13.25	0.100
T-C (mg/dL)	168.74 ± 30.31	169.37 ± 27.57	0.687
TG (mg/dL)	109.57 ± 58.75	109.37 ± 55.41	0.951
HDL-C (mg/dL)	45.16 ± 8.42	47.28 ± 8.72	< 0.001
LDL-C (mg/dL)	101.67 ± 26.99	100.22 ± 24.24	0.297
Alcohol consumption (%)	192 (26.34%)	147 (23.22%)	0.207
Smoking status (%)	101 (13.85%)	47 (7.42%)	< 0.001
Household income < 2nd quartile (%)	79 (10.84%)	88 (13.90%)	0.102
Rural residential area (%)	114 (15.64%)	104 (16.43%)	0.947
Physical activity (%)	282 (38.68%)	210 (33.18%)	0.040

*SDS* standard deviation score, *WC* waist circumference, *BMI* body mass index, *TMI* tri-ponderal mass index, *SBP* systolic blood pressure, *DBP* diastolic blood pressure, *T-C* total cholesterol, *TG* triglyceride, *HDL-C* high-density lipoprotein cholesterol, *LDL-C* low-density lipoprotein cholesterol.

### Clinical characteristics and cardiometabolic risks based on the TMI obesity classification

Overall, the means of cardiometabolic risks tended to increase with increasing severity of obesity according to the TMI classification. However, there were sex differences in these cardiometabolic risk factors. In male participants, the means of weight SDS, WC SDS, BMI SDS, SBP, DBP, TG, HDL-c, and LDC-c increased with increasing severity of obesity. However, fasting glucose (*p* = 0.013) was included, but the LDL-c levels (*p* = 0.561) did not differ by obesity classification in female participants. The clinical characteristics and cardiometabolic risks are summarized in Table 2.

**Table 2:** Clinical characteristics of the study population according to obesity class (*n* = 1362).

Male participants					
	Overweight	Class I obesity	Class II obesity	Class III obesity	*P*
	(*n* = 505)	(*n* = 141)	(*n* = 56)	(*n* = 27)	
Age (years)	14.14 ± 2.50	14.34 ± 2.36	14.79 ± 2.61	14.69 ± 2.06	0.202
Height SDS	0.32 ± 1.07	0.38 ± 0.98	0.11 ± 1.09	− 0.11 ± 1.14	0.079
Weight SDS	1.50 ± 0.79	2.21 ± 0.78^a^	2.61 ± 0.86^b,c^	3.13 ± 0.91^d,e,f^	< 0.001
WC SDS	1.11 ± 0.54	1.63 ± 0.50^a^	2.15 ± 0.67^b,c^	2.69 ± 1.04^d,e,f^	< 0.001
BMI SDS	1.69 ± 0.44	2.52 ± 0.43^a^	3.17 ± 0.54^b,c^	3.92 ± 0.57^d,e,f^	< 0.001
TMI SDS	1.30 ± 0.17	1.78 ± 0.09^a^	2.11 ± 0.10^b,c^	2.53 ± 0.16^d,e,f^	< 0.001
SBP (mmHg)	113.56 ± 9.95	115.69 ± 10.91	119.27 ± 12.91^b^	120.81 ± 11.67^d^	0.002
DBP (mmHg)	67.84 ± 9.45	69.91 ± 9.65	71.39 ± 10.04	72.33 ± 10.99	0.016
Glucose (mg/dL)	92.10 ± 7.54	92.62 ± 6.43	92.48 ± 7.47	94.63 ± 7.74	0.336
T-C (mg/dL)	167.09 ± 29.51	169.88 ± 33.76	176.71 ± 27.74	177.22 ± 28.44	0.052
TG (mg/dL)	105.30 ± 56.05	117.24 ± 61.13	117.73 ± 70.03	132.33 ± 62.45	0.017
HDL-C (mg/dL)	45.76 ± 8.59	44.48 ± 8.26	43.02 ± 7.35	41.99 ± 6.56	0.012
LDL-C (mg/dL)	100.27 ± 26.31	101.95 ± 29.05	110.15 ± 27.21	108.77 ± 25.12	0.032

Female participants					
	(*n* = 410)	(*n* = 118)	(*n* = 61)	(*n* = 44)	
Age (years)	14.18 ± 2.51	14.33 ± 2.54	14.43 ± 2.87	15.39 ± 2.12^d^	0.026
Height SDS	0.14 ± 1.03	− 0.02 ± 1.14	− 0.27 ± 1.42^b^	0.14 ± 1.41	0.042
Weight SDS	1.30 ± 0.73	1.75 ± 0.71^a^	2.00 ± 0.99^b^	3.13 ± 0.82^d,e,f^	< 0.001
WC SDS	0.92 ± 0.65	1.47 ± 0.61^a^	1.75 ± 0.97^b^	2.87 ± 1.18^d,e,f^	< 0.001
BMI SDS	1.49 ± 0.43	2.13 ± 0.37^a^	2.60 ± 0.53^b,c^	3.73 ± 0.56^d,e,f^	< 0.001
TMI SDS	1.32 ± 0.18	1.78 ± 0.09^a^	2.12 ± 0.09^b,c^	2.61 ± 0.32^d,e,f^	< 0.001
SBP (mmHg)	107.03 ± 9.26	107.14 ± 11.23	111.36 ± 9.55^b,c^	114.80 ± 11.09^d,e^	< 0.001
DBP (mmHg)	65.98 ± 7.94	66.31 ± 8.81	69.87 ± 8.35^b,c^	72.34 ± 9.78^d,e^	< 0.001
Glucose (mg/dL)	90.13 ± 6.78	93.92 ± 25.94^a^	92.18 ± 10.57	94.59 ± 10.55	0.013
T-C (mg/dL)	168.46 ± 27.28	171.90 ± 26.45	169.51 ± 30.85	170.95 ± 28.84	0.663
TG (mg/dL)	104.84 ± 51.88	119.28 ± 62.92	110.75 ± 57.06	123.09 ± 59.57	0.026
HDL-C (mg/dL)	48.17 ± 8.67	46.56 ± 8.17	46.26 ± 9.16	42.31 ± 8.38^d,e^	< 0.001
LDL-C (mg/dL)	99.31 ± 23.93	101.48 ± 23.11	101.10 ± 28.08	104.03 ± 24.62	0.561

Data are presented as the means ± standard deviation (SD).

*SDS* standard deviation score, *WC* waist circumference, *BMI* body mass index, *TMI* tri-ponderal mass index, *SBP* systolic blood pressure, *DBP* diastolic blood pressure, *T-C* total cholesterol, TG triglyceride, *HDL-C* high-density lipoprotein cholesterol, *LDL-c* low-density lipoprotein cholesterol.

Class I obesity was defined as a TMI ≥ 95th percentile and < 120% of 95th percentile TMI.

Class II obesity was defined as a TMI ≥ 120% of the 95th percentile and < 140% of the 95th percentile TMI.

Class III obesity was defined as a TMI ≥ 140% of the 95th percentile of TMI.

Statistical significance (*P*-value) was determined using analyses of variance (ANOVAs) according to sex.

^a^: Statistical significance was determined using ANOVA between overweight and Class I obesity with Bonferroni’s *post-hoc* analysis.

^b^: Statistical significance was determined using ANOVA between overweight and Class II obesity with Bonferroni’s *post-hoc* analysis.

^c^: Statistical significance was determined using ANOVA between Class I obesity and Class II obesity with Bonferroni’s *post-hoc* analysis.

d: Statistical significance was determined using ANOVA between overweight and Class III obesity with Bonferroni’s *post-hoc* analysis.

^e^: Statistical significance was determined using ANOVA between Class I obesity and Class III obesity with Bonferroni’s *post-hoc* analysis.

^f^: Statistical significance was determined using ANOVA between Class II obesity and Class III obesity with Bonferroni’s *post-hoc* analysis.

### Adjusted means and standard errors of cardiometabolic risks according to the TMI obesity classification

As the severity of obesity increased, cardiometabolic risks were also increased but there were sex differences in metabolic syndrome components such as fasting glucose levels, T-C, and LDL-c. With increasing severity of obesity, the means of WC SDS, BMI SDS, SBP, DBP, T-C, TG, HDL-c, and LDL-c increased in male participants. Similarly, WC SDS, BMI SDS, SBP, DBP, fasting glucose level, TG and HDL-c were significantly increased in female participants, but T-C and LDL-c were not influenced by the severity of obesity after adjusting for age, alcohol consumption, smoking status, household income, rural residential area, physical activity, and diagnosis of type 2 diabetes mellitus (T2DM), hypertension, and dyslipidemia (Table 3).

**Table 3:** Adjusted means and standard errors of cardiometabolic risks according to obesity class in study population (*n* = 1362).

Male participants					
	Overweight	Class I obesity	Class II obesity	Class III obesity	*P* for trend
	(*n* = 505)	(*n* = 141)	(*n* = 56)	(*n* = 27)	
WC SDS	1.12 ± 0.03	1.62 ± 0.05^a^	2.13 ± 0.08^b,c^	2.67 ± 0.11^d,e,f^	< 0.001
BMI SDS	1.70 ± 0.02	2.52 ± 0.04^a^	3.13 ± 0.06^b,c^	3.90 ± 0.08^d,e,f^	< 0.001
SBP (mmHg)	113.71 ± 0.44	115.58 ± 0.83	118.54 ± 1.31^b^	119.99 ± 1.89^d^	< 0.001
DBP (mmHg)	67.92 ± 0.41	69.92 ± 0.77	70.87 ± 1.22	71.83 ± 1.76	< 0.001
Glucose (mg/dL)	92.05 ± 0.32	92.66 ± 0.61	92.76 ± 0.97	94.70 ± 1.39	0.062
T-C (mg/dL)	167.18 ± 1.35	169.84 ± 2.55	176.26 ± 4.06	176.62 ± 5.84	0.012
TG (mg/dL)	105.53 ± 2.59	117.10 ± 4.90	116.67 ± 7.79	130.92 ± 11.22	0.004
HDL-C (mg/dL)	45.70 ± 0.36	44.54 ± 0.69	43.32 ± 1.10	42.24 ± 1.58	0.003
LDL-C (mg/dL)	100.38 ± 1.20	101.88 ± 2.27	109.61 ± 3.61	108.19 ± 5.19	0.012

Female participants					
	(*n* = 410)	(*n* = 118)	(*n* = 61)	(*n* = 44)	
WC SDS	0.93 ± 0.04	1.47 ± 0.07^a^	1.74 ± 0.09^b^	2.84 ± 0.11^d,e,f^	< 0.001
BMI SDS	1.50 ± 0.02	2.13 ± 0.04^a^	2.58 ± 0.05^b,c^	3.64 ± 0.06^d,e,f^	< 0.001
SBP (mmHg)	107.09 ± 0.48	107.18 ± 0.89	111.25 ± 1.24^b,c^	114.35 ± 1.49^d,e^	< 0.001
DBP (mmHg)	66.06 ± 0.40	66.24 ± 0.75	69.83 ± 1.04^b,c^	71.81 ± 1.25^d,e^	< 0.001
Glucose (mg/dL)	90.07 ± 0.65	94.05 ± 1.21^a^	92.04 ± 1.68	95.00 ± 2.02	0.004
T-C (mg/dL)	168.55 ± 1.36	172.03 ± 2.54	169.37 ± 3.53	169.87 ± 4.24	0.550
TG (mg/dL)	104. 59 ± 2.72	119.50 ± 5.07	111.00 ± 7.04	124.53 ± 8.46	0.009
HDL-C (mg/dL)	48.16 ± 0.43	46.56 ± 0.79	46.22 ± 1.10	42.49 ± 1.32d	< 0.001
LDL-C (mg/dL)	99.47 ± 1.19	101.58 ± 2.23	100.95 ± 3.09	102.47 ± 3.72	0.333

Data are presented as the means ± standard errors (SE).

*SDS* standard deviation score, *WC* waist circumference, *BMI* body mass index, *TMI* tri-ponderal mass index, *SBP* systolic blood pressure, *DBP* diastolic blood pressure, *T-C* total cholesterol, *TG* triglyceride, *HDL-C* high-density lipoprotein cholesterol, *LDL-c* low-density lipoprotein cholesterol.

Class I obesity was defined as a TMI ≥ 95th percentile and < 120% of the 95th percentile TMI.

Class II obesity was defined as a TMI ≥ 120% of 95th percentile and < 140% of the 95th percentile TMI.

Class III obesity was defined as a TMI ≥ 140% of 95th percentile of TMI.

Adjusted means were calculated using an analysis of covariance (ANCOVA) after adjustment for age, alcohol consumption, smoking status, household income, rural residential area, physical activity, and diagnosis of type 2 diabetes mellitus (T2DM), hypertension, and dyslipidemia according to sex.

^a^: Statistical significance was determined using ANCOVA between overweight and Class I obesity with Bonferroni’s *post-hoc* analysis.

^b^: Statistical significance was determined using ANCOVA between overweight and Class II obesity with Bonferroni’s *post-hoc* analysis.

^c^: Statistical significance was determined using ANCOVA between Class I obesity and Class II obesity with Bonferroni’s *post-hoc* analysis.

d: Statistical significance was determined using ANCOVA between overweight and Class III obesity with Bonferroni’s *post-hoc* analysis.

^e^: Statistical significance was determined using ANCOVA between Class I obesity and Class III obesity with Bonferroni’s *post-hoc* analysis.

^f^: Statistical significance was determined using ANCOVA between Class II obesity and Class III obesity with Bonferroni’s *post-hoc* analysis.

### Prevalence of cardiometabolic risks according to the TMI obesity classification

**Figure 1:**
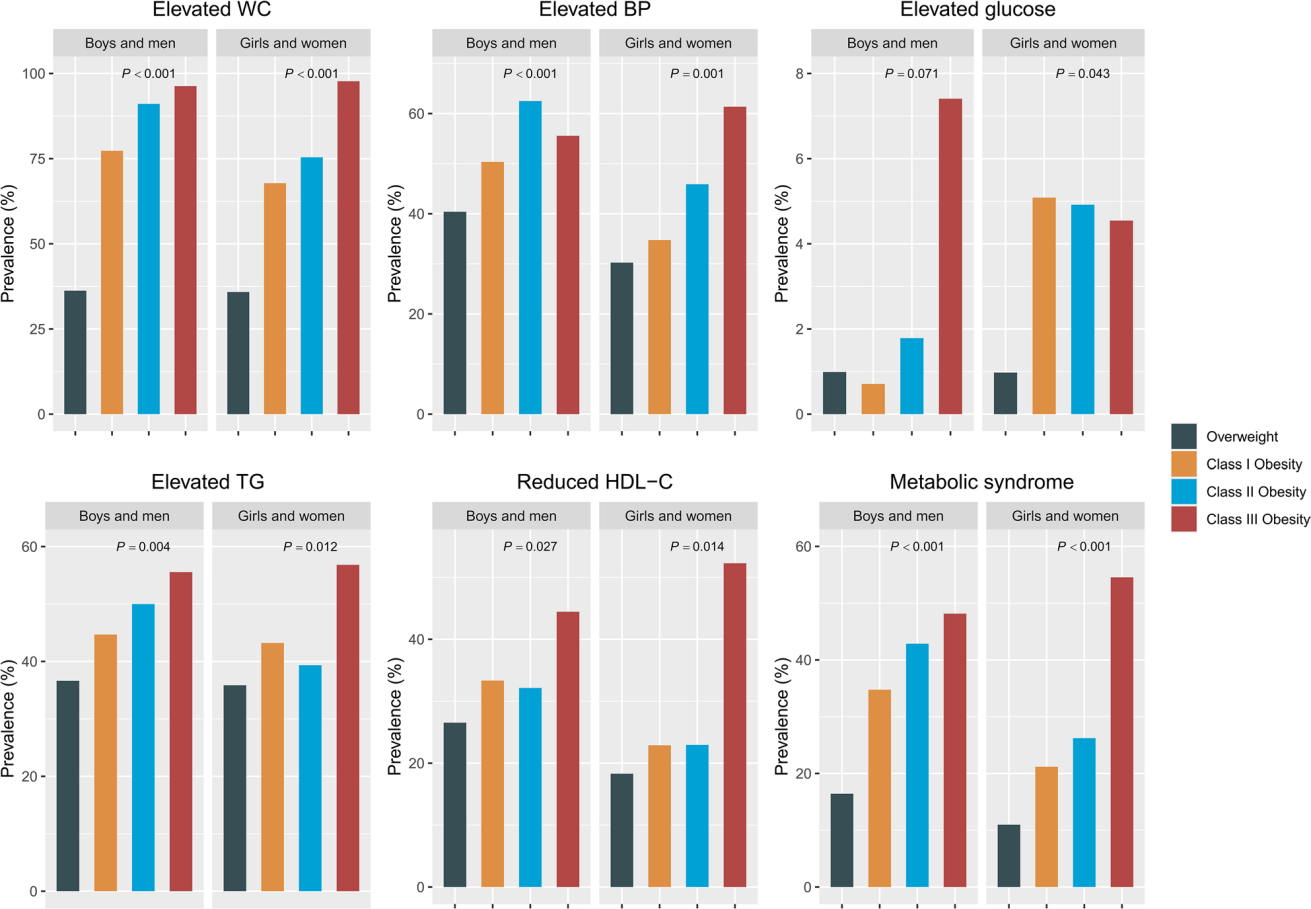
The prevalence of metabolic syndrome and its components according to obesity class (n = 1362). Statistical significance was determined using chi-square tests. WC, waist circumference; BP, blood pressure; TG, triglycerides; HDL-c, high-density lipoprotein cholesterol.

As the obesity classification (overweight, Class I obesity, Class II obesity, Class III obesity) increased, the prevalence of metabolic syndrome was increased in both males and females. The prevalence of cardiometabolic risk factors, including elevated WC, elevated BP, elevated TG (and low HDL-c were also increased with the severity of obesity (overweight, Class I obesity, Class II obesity, and Class III obesity, respectively) in both sexes. The incidence of abnormal fasting glucose levels tended to increase in both males and females, but the increase was only significant in female participants (1.0%, 0.7%, 1.8%, and 7.4%, *p* = 0.071 for males and 1.0%, 5.1%, 4.9%, and 4.5%, *p* = 0.043 for females) (Fig. 1).

### Increased odds ratios of cardiometabolic risk factors according to the TMI obesity classification

The ORs for metabolic syndrome and its components increased as the severity of obesity increased, as summarized in Table 4. Model 1 was a logistic regression analysis according to the obesity class that did not adjust for other variables. In this model, as the severity of obesity increased, the OR of metabolic syndrome increased by 2.46 (95% CI 0.99–1.84) for Class I obesity, 3.19 (95% CI 2.09–4.89) for Class II obesity and 6.69 (95% CI 4.05–11.05) for Class III obesity. Increased ORs of elevated TG and lower HDL-c were not significant for Class II obesity; however, all ORs of the cardiometabolic risk factors were significantly increased in Class III obesity. Model 2 was a multiple logistic regression analysis that adjusted for age and sex. Model 3 was similar to Model 2 and additionally adjusted for alcohol consumption, smoking status, household income, rural residential area, physical activity, and diagnosis of T2DM, hypertension, and dyslipidemia. In both Model 2 and Model 3, adjusted ORs for elevated WC, elevated BP, elevated fasting glucose levels, elevated TG and lower HDL-c TG increased with the severity of obesity. Moreover, adjusted ORs for metabolic syndrome components were considerably increased. The magnitude of the ORs was similar in the three models. In the gender-separated logistic regression analysis, increased ORs for metabolic syndromes and its components were observed but statistical difference between males and females was not found. Data summarized in Supplementary Table 1.

**Table 4:** Unadjusted and adjusted odds ratios (ORs) for metabolic syndrome and its components according to obesity class in the study population (*n* = 1362).

	Overweight	Class I obesity	Class II obesity	Class III obesity
	(*n* = 915)	(*n* = 259)	(*n* = 117)	(*n* = 71)
**Model 1**				
Elevated WC	Reference	4.79 (3.53–6.50)	8.60 (5.22–14.17)	61.16 (14.90–251.08)
Elevated BP	Reference	1.36 (1.03–1.81)	2.09 (1.42–3.08)	2.59 (1.58–4.24)
Elevated glucose	Reference	2.80 (1.03–7.58)	3.56 (1.08–11.76)	6.01 (1.80–20.03)
Elevated TG	Reference	1.38 (1.04–1.83)	1.40 (0.95–2.07)	2.27 (1.39–3.69)
Low HDL-C	Reference	1.35 (0.99–1.84)	1.27 (0.82–1.96)	3.28 (2.01–5.36)
MetS	Reference	2.46 (1.77–3.41)	3.19 (2.09–4.89)	6.69 (4.05–11.05)
**Model 2**				
Elevated WC	Reference	4.84 (3.55–6.59)	8.66 (5.23–14.34)	58.72 (14.27–241.59)
Elevated BP	Reference	1.36 (1.03–1.81)	2.14 (1.44–3.17)	2.71 (1.64–4.46)
Elevated glucose	Reference	2.77 (1.02–7.52)	3.37 (1.02–11.16)	5.26 (1.55–17.85)
Elevated TG	Reference	1.38 (1.04–1.83)	1.41 (0.96–2.09)	2.30 (1.41–3.76)
Low HDL-C	Reference	1.34 (0.98–1.84)	1.26 (0.81–1.96)	3.33 (2.02–5.48)
MetS	Reference	2.47 (1.77–3.45)	3.22 (2.09–4.97)	6.87 (4.11–11.49)
**Model 3**				
Elevated WC	Reference	4.85 (3.56–6.62)	8.75 (5.27–14.51)	61.34 (14.88–252.81)
Elevated BP	Reference	1.37 (1.03–1.82)	2.18 (1.47–3.23)	2.74 (1.66–4.53)
Elevated glucose	Reference	2.85 (1.04–7.76)	3.36 (1.00–11.24)	4.87 (1.41–16.78)
Elevated TG	Reference	1.39 (1.05–1.84)	1.41 (0.95–2.08)	2.28 (1.39–3.37)
Low HDL-C	Reference	1.35 (0.98–1.84)	1.27 (0.82–1.97)	3.28 (1.99–5.41)
MetS	Reference	2.51 (1.80–3.50)	3.27 (2.11–5.05)	6.81 (4.07–11.41)

*WC* waist circumference, *BP* blood pressure, *TG* triglyceride, *HDL-c* high-density lipoprotein cholesterol, *MetS* metabolic syndrome.

Class I obesity was defined as a TMI ≥ 95th percentile and < 120% of the 95th percentile TMI.

Class II obesity was defined as a TMI ≥ 120% of the 95th percentile and < 140% of 95th percentile TMI.

Class III obesity was defined as a TMI ≥ 140% of the 95th percentile of TMI.

Model 1: Statistical significance was determined using logistic regression analysis with no adjustment according to obesity class.

Model 2: Statistical significance was determined using multiple logistic regression analysis after adjustment for age and sex according to obesity class.

Model 3: Statistical significance was determined using multiple logistic regression analysis after adjustment for age, sex, alcohol consumption, smoking status, household income, rural residential area, physical activity, and diagnosis of type 2 diabetes mellitus (T2DM), hypertension, and dyslipidemia according to obesity class.

## Discussion

We found that the metabolic risk factors that accompany obesity were clearly associated with the severity of obesity, as classified by TMI. As the severity of obesity increased, body weight, BMI SDS, and the degree of abdominal obesity increased. Additionally, the incidence of metabolic syndromes was also significantly higher and the ORs of metabolic syndrome and its components significantly increased as the severity of obesity increased, which is important clinical evidence that the TMI-based classifications can guide patient prognosis and predict complications.

The classification of obesity has been emphasized because the severity of obesity is closely associated with the morbidity of metabolic syndromes in children^12,18,19^, which substantially increases the risk of future diseases in adulthood such as T2DM and CVD^20–25^. Moreover, age at the onset of obesity, as well as the time period that obesity lasts, influences metabolic syndrome in adolescence^26^. Therefore, early diagnosis, precise classifications, and appropriate interventions are important for the prevention of subsequent morbidity and mortality due to cardiometabolic diseases. TMI may more accurately determine body fat composition than BMI. As suggested in earlier studies, adolescent weight scales with height at a power of at least 2.5, and at 3 to 3.5 between 10 and 15 years of age^27,28^. More recently, TMI has been proposed as an alternative to BMI z-scores to estimate body adiposity, because TMI is both more stable with age and more accurate than BMI z-scores^16,29^. BMI SDS- and z score- based classification has suffered from a high rate of misclassification. In our previous study that classified subjects according to BMI, 49.3% of the BMI classified “obese” patients were reclassified as overweight based on TMI, and 2.6% of these obese patients were reclassified as normal weight, in which we suggested that TMI was better at discriminating changes in metabolic risk factors than BMI^17^. However, TMI is based on the statistical distribution of body fat (as is the BMI z score) rather than on health risks; therefore, the relationship between adolescent adiposity and health risk factors required clarification.

In a previous study that classified obesity based on BMI, overweight categories were defined according to age- and sex-specific percentiles, as overweight (85th ≤ BMI < 95th percentile), Class I obesity (95th percentile ≤ BMI < 120% of the 95th percentile), Class II obesity (120% of the 95th percentile ≤ BMI < 140% of the 95th percentile or BMI ≥ 35 kg/m^2^, whichever was lower), and Class III obesity (BMI ≥ 140% of the 95th percentile or BMI ≥ 40 kg/m^2^, whichever was lower)^18^. We created the same four classifications using TMI according to percentiles by sex and age, but the specific cutoff values were not applied because there was variability in the threshold values between age, races, and regions^17,30^.

Compared to a previous report in which the prevalence of overweight and Class I, II, and III obesity in 10- to 18-years old subjects of KNHANES 2007–2014 was 5.6%, 6.2%, 5.9%, and 0.1%, respectively^12^, we observed that the prevalence of these categories in the same age range of subjects of KNHANES 2007–2017 data was 12.6%, 3.6%, 1.6%, and 1%, respectively; our data exhibited a sequential decrease in the TMI classifications. This discrepancy is possibly due to an increase in overweight children over the study period, but rather it is more likely due to increased diagnostic accuracy. The prevalence of metabolic syndrome, as well as cardiometabolic risk factors, was also higher in our TMI classifications than that in the previously reported BMI-based classifications of the same KNHANES age group. For instance, the prevalence of TG > 150 mg/dL in the overweight, Class I, Class II, and Class III obesity categories was 15.3%, 16.7%, 26.5%, and 30.9%, respectively, in the BMI classifications. However, the prevalence found with our TMI-based classifications was 36.6%, 44.7%, 50.0%, and 55.6%, respectively, for males and 35.9%, 43.2%, 39.3%, and 56.8%, respectively, for females. The ratios of elevated BP, impaired fasting glucose levels, and low HDL-c were also higher with the TMI classifications. Although both studies were based on the same age group and population, the prevalence of metabolic syndrome markedly differed for each subgroup. The effect of the additional investigation period (an additional of three years of data in our study compared to the previous study) cannot be excluded. However, these findings may imply that TMI more accurately predicts metabolic syndrome risk as it better reflects body adiposity^16,31^.

Interestingly, we found sex differences in the prevalence of metabolic syndrome: the rate of metabolic syndrome increased with severity of obesity starting from Class I obesity in boys but tended to rapidly increase at Class III obesity in girls. Similarly, obesity was accompanied by an increase in cardiometabolic risk factors, but the exact risk factors differed between boys and girls. In both sexes, the BP, TG, HDL-c, and fasting blood glucose levels increased significantly with the obesity classification, but lipid profiles, including T-C and LDL-c levels, were not affected in girls. ﻿﻿Sex difference in the prevalence of metabolic risk factors remain debatable because only a limited number of studies have reported these findings^32,33^. However, our results are consistent with those of previous studies in which cardiometabolic risk factors developed earlier in boys than in girls in the United States^18^. Such differences might be due to hormonal differences or differences in body fat percentage according to the onset of puberty. However, the risk of metabolic syndrome increased from the initial stage of obesity in boys; thus, the severity of obesity and risk evaluations should be determined depending on sex.

Our study was limited in that it was a cross-sectional analysis; therefore, we could not determine the temporal association of metabolic syndrome with the severity of obesity. A large cohort longitudinal study is needed to clarify the impact of the severity of obesity on future cardiometabolic risks. Although body adiposity also depends on height as well as sexual maturity, we did not assess the effect of a puberty on the severity of obesity as the KNHANES did not collect data on the degree of sexual maturation. It is possible that overweight children enter puberty earlier and are taller than lean individuals. Future studies should include the pubertal status and determine actual body adiposity using the dual-energy X-ray absorptiometry (DXA), which may determine the accuracy of the classifications. Finally, we found the gender differences in the prevalence of metabolic syndrome according to the severity of obesity; however, the differences of ORs were not statistically significant in the gender-separated logistic regression analysis (supplementary Table 1). This seems to be due to the relatively small number of class III obesity in the analyzed subjects, therefore expanded analysis with larger population data will be needed in further studies.

The increase in the OR for metabolic syndrome in severe obesity was closely associated with the prevalence of metabolic syndrome and its components even after adjusting for several confounding variables. In particular, Class III obesity subjects had a considerably higher prevalence of metabolic syndrome. Our results showed that the TMI obesity classification is useful in predicting the prevalence and risk of metabolic syndrome according to increases in the severity of obesity. Therefore, clinical application of classification of childhood obesity according to TMI and early detection for metabolic syndrome with customized treatment strategy might be helpful to reduce the risks of childhood cardiometabolic complications as well as advanced adulthood risk for complications of obesity.

## Materials and methods

This study was based on data from the KNHANES from 2007 to 2018. The KNHANES is a cross-sectional, nationally representative survey that is conducted annually by the Korean Centers for Disease Control and Prevention (KCDC). The survey consists of a health questionnaire, health examination, and nutritional assessment. The KNHANES uses a stratified and multistage probability sampling design to select household units for inclusion; individuals are randomly selected. The database is publicly available on the KNHANES website (http://knhanes.cdc.go.kr). All KNHANES subjects provided informed consent at the time of data collection and the survey protocols were approved by the KCDC Institutional Review Board. Details of the KNHANES have previously been described^34^. This study was approved by the Institutional Review Board of Hallym University Chuncheon Sacred Heart Hospital (IRB No. CHUNCHEON 2022–03-014). All methods were performed in accordance with the relevant guidelines and regulations. The IRB waived the requirement for written informed consent due to the retrospective study design.

### Subjects

**Figure 2:**
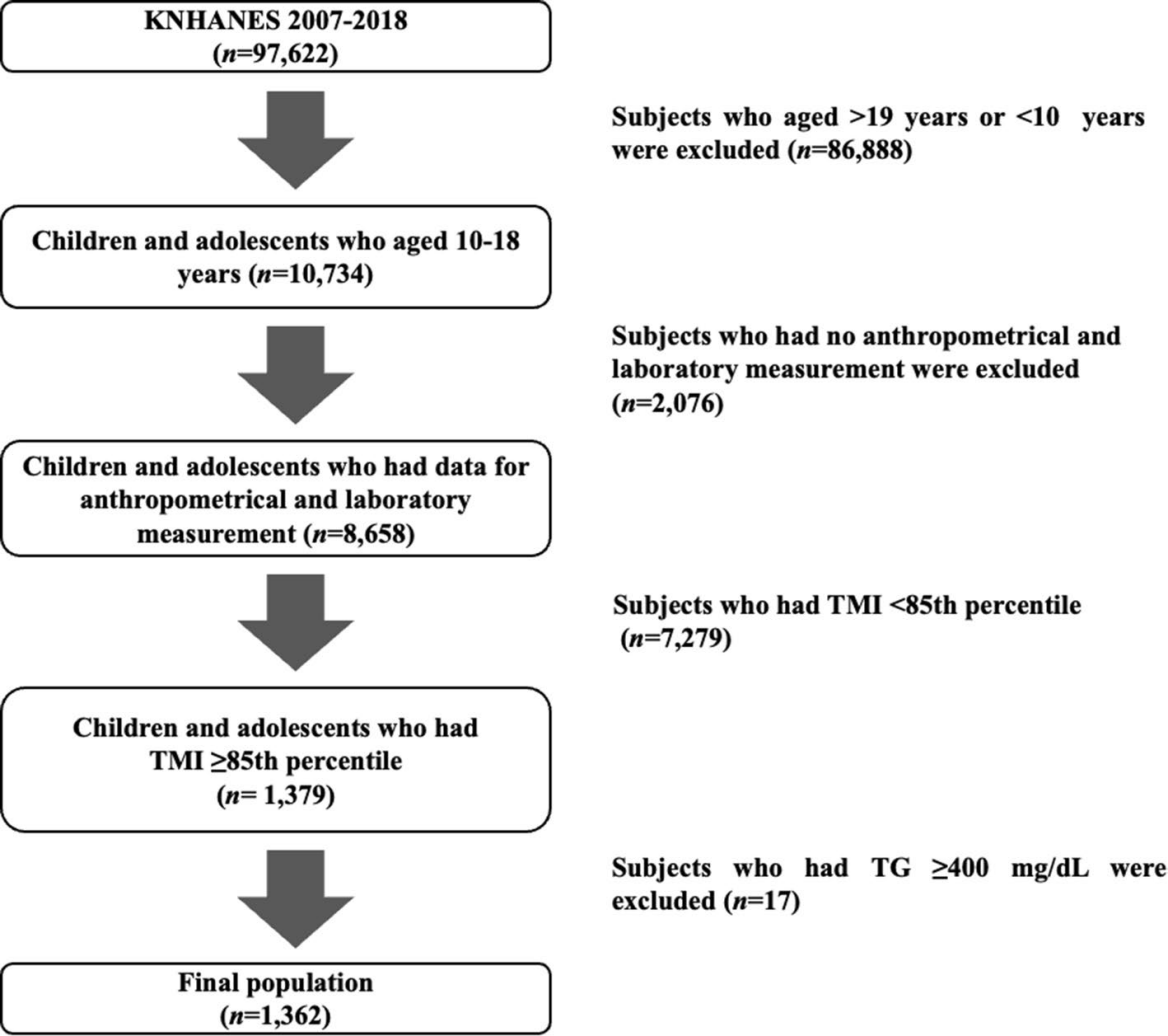
Flow chart of the study population.

The KNHANES 2007–2018 data included a total of 97,622 subjects; of these, 10,734 participants aged 10 to 18 years were included in the preliminary analysis. Subjects less than 95th percentile of TMI who considered normal weight were excluded from the analysis. After applying the exclusion criteria (no anthropometric or laboratory data, TMI < 85th percentile and TG ≥ 400 mg/dL), 1362 participants (729 boys, 633 girls) remained and were included in the final analysis (Fig. 2).

### Anthropometric and laboratory measurements.

Anthropometric data were collected by trained experts according to standardized protocols. Height was measured to the nearest 0.1 cm using an electronic stadiometer Seca 225 (Seca, Hamburg, Germany). Weight was measured to the nearest 0.1 kg using GL-6000–20 calibrated standard beam scales (G-tech, Seoul, Korea). WC was measured to the nearest 0.1 cm at the midline between the lower rib margin and iliac crest. BMI and TMI were calculated as weight (kg)/height (m^2^) and weight (kg)/height (m^3^), respectively. SDSs were calculated for height, weight, BMI, WC, and TMI with LMS methods using the 2017 and 2020 Korean references^3,17^. For the accurate age- and sex- adjusted estimates, anthropometric measurements were converted to SDS and expressed as the number of SDs above and below the mean for each subject. Blood pressure was measured 3 times on the right upper arm using a calibrated sphygmomanometer (Baumanometer Desk model 0320, Baum, NY, USA) with an appropriate-sized cuff while the subject was seated. ﻿Each measurement was performed at 2-min intervals, and the mean BP of the last two measurements was used for analysis. Blood samples for laboratory evaluation were collected after at least 8 h of fasting. All samples were immediately centrifuged, transported to a central laboratory (NeoDin Medical Institute, Seoul, Korea) and analyzed within 24 h. Serum levels of total cholesterol (T-C), high-density lipoprotein cholesterol (HDL-c), TG, and glucose levels were measured enzymatically using a Hitachi 7600 automatic analyzer (Hitachi, Tokyo, Japan). LDL-c was calculated with Friedewald’s equation^35^.

### Lifestyle parameters and socioeconomic status.

Socioeconomic status (household income, residential area) and lifestyle-related parameters (smoking status, alcohol consumption, physical activity) were collected by questionnaires. Household income was categorized into quartiles (1: low, 2: middle low, 3: middle high, 4: high). Residence areas were divided into urban or rural areas. Smoking status was defined by whether individuals had smoked more than five packs of cigarettes in their lifetime. Alcohol consumption was defined as the consumption of at least two alcoholic beverages per month during the previous year. Subjects were considered to exercise if they met any of one of the following criteria: (1) intense physical activity for 30 min at least three days/week, (2) moderate physical activity for 30 min at least five days/week, or (3) walking for 30 min at least five days/week.

### Study criteria

Overweight individuals were defined as having a TMI ≥ 85th percentile and < 95th percentile. Obesity was defined as ≥ 95th percentile TMI and was further separated into 3 classes according to the increased cardiometabolic risks associated with increased TMI: Class I was defined as the 95th percentile ≤ TMI < 120% of the 95th percentile; Class II was defined as ≥ 120% of the 95th percentile and < 140% of the 95th percentile; and Class III was defined as ≥ 140% of the 95th percentile.

### Definitions of the cardiometabolic risk factors

Modified criteria for cardiometabolic risk factors were adapted from the National Cholesterol Education Program Adult Treatment Panel III (NCEP-ATP III) as follows^36^: (1) WC ≥ 90th percentile for age and sex according to the 2017 Korean growth chart^3^, (2) blood pressure (BP), systolic blood pressure (SBP) or diastolic blood pressure (DBP) ≥ 90th percentile for age- and sex-matched reference data from the Korean population^3^ or treatment with antihypertensive medication, (3) fasting blood glucose ≥ 100 mg/dL or treatment for type 2 diabetes mellitus (T2DM), (4) elevated TG ≥ 110 mg/dL or treatment for dyslipidemia, and (5) low HDL-c < 40 mg/dL. Metabolic syndrome was defined as the presence of at least three of the five criteria. T2DM was diagnosed in patients that satisfied at least one of the following criteria: (1) self-report on a questionnaire consisting of yes or no answers, (2) current medication or insulin use to manage T2DM, or (3) a fasting glucose level ≥ 126 mg/dL during the survey period.

### Statistical analysis

All statistical analyses in this study were performed using R statistical package version 3.5.1 (The R Foundation for Statistical Computing, Vienna, Austria). Percentile curves of TMI were estimated by the LMS model to fit smoothed L (skew), M (median), and S (coefficient of variation) curves in the General Additive Model for Location Scale and Shape (GAMLSS) package version 4.2.6. of the R statistical package. Details have been described in our previous study^17^. The data consisted of continuous variables and categorical variables; these variables are presented as the mean ± standard deviation (SD) and frequencies or percentages (%), respectively. Student’s t-tests and chi-square (χ2) tests were used to compare the means of demographic and biochemical characteristics and categorical variables between boys and girls. The anthropometric and laboratory data of body composition according to obesity classification (overweight or Class I, Class II and Class III obesity) were compared using analysis of variance (ANOVAs) with Bonferroni corrections. The adjusted mean values of the cardiometabolic risk factors were compared using analysis of covariance (ANCOVA) followed by Bonferroni’s post-hoc test after adjusting for sex, age, BMI SDS, alcohol consumption, smoking status, physical activity, residential area, household income, and diagnosis of hypertension, T2DM, and dyslipidemia. We estimated the adjusted odds ratio and 95% confidence intervals (CIs) for metabolic syndrome among the adiposity classifications by multiple logistic regression analysis. The estimated odds were calculated by Model 1, Model 2, and Model 3; each represented adjustments for differential covariates including sex, age, BMI SDS, alcohol consumption, smoking status, physical activity, residential area, household income, and diagnosis of hypertension, T2DM, and dyslipidemia. *P* < 0.05 was considered indicative of statistical significance.

## Supplementary Information

Supplementary Information.

## Data Availability

The data that support the findings of this study are available in [the KNHANES website] at [https://knhanes.kdca.go.kr]. The data that support the findings of this study are available from the corresponding author, [Shim YS], upon reasonable request.

## Abbreviations

ANCOVA: Analysis of covariance
ALT: Alanine aminotransferase
AST: Aspartate aminotransferase
BP: Blood pressure
BMI: Body mass index
DBP: Diastolic blood pressure
CI: Confidence interval
CVD: Cardiovascular disease
HDL-C: High-density lipoprotein cholesterol
KNHANES: The Korea National Health and Nutrition Examination Survey
LDL-C: Low-density lipoprotein cholesterol
MetS: Metabolic syndrome
ORs: Odds ratios
SBP: Systolic blood pressure;
SDS: Standard deviation scores
TMI: Tri-ponderal mass index
T2DM: Type 2 diabetes mellitus
TG: Triglyceride
T-C: Total cholesterol
WHO: World Health Organization
WC: Waist circumference
