# Supplementary material for: Metabolic risk assessment in children and adolescents using the tri-ponderal mass index
Source: Sci Rep. 2022 Jun 16;12:10094. doi: 10.1038/s41598-022-13342-7 (PMC9203500; doi:10.1038/s41598-022-13342-7)
Supplement: Supplementary file 1 — Supplementary Information. [file 41598_2022_13342_MOESM1_ESM.docx]

**Supplement Table 1.**

Odds ratios (ORs) for metabolic syndrome and its components by gender differences.

| Boys | | | | |
| --- | --- | --- | --- | --- |
|  | Model 1 | | | |
|  | Overweight | Class I obesity | Class II obesity | Class III obesity |
|  | (*n*=505) | (*n*=141) | (*n*=56) | (*n*=27) |
| Elevated WC | Reference | 5.99 (3.88-9.25)* | 17.95 (7.04-45.77)* | 45.75 (6.16-339.62)* |
| Elevated BP | Reference | 1.50 (1.03-2.18)* | 2.46 (1.39-4.35)* | 1.84 (0.85-4.02) |
| Elevated glucose | Reference | 0.71 (0.08-6.16) | 1.82 (0.21-15.84) | 8.00 (1.48-43.28)* |
| Elevated TG | Reference | 1.40 (0.96-2.04) | 1.73 (0.99-3.01) | 2.16 (0.99-4.72) |
| Elevated HDL-C | Reference | 1.38 (0.93-2.07) | 1.31 (0.72-2.38) | 2.21 (1.01-4.85)* |
| MetS | Reference | 2.71 (1.78-4.12)* | 3.81 (2.14-6.81)* | 4.72 (2.14-10.41)* |
| Model 2 | Model 2 | | | |
| Elevated WC | Reference | 6.00 (3.88-9.27)* | 17.44 (6.82-44.57)* | 44.54 (5.99-331.06)* |
| Elevated BP | Reference | 1.48 (1.01-2.15)* | 2.33 (1.31-4.15)* | 1.76 (0.80-3.85) |
| Elevated glucose | Reference | 0.72 (0.08-6.18) | 1.83 (0.21-16.10) | 8.06 (1.48-43.96)* |
| Elevated TG | Reference | 1.38 (0.95-2.02) | 1.66 (0.95-2.90) | 2.09 (0.96-4.58) |
| Elevated HDL-C | Reference | 1.36 (0.91-2.05) | 1.20 (0.65-2.19) | 2.10 (0.95-4.64) |
| MetS | Reference | 2.72 (1.77-4.16)* | 3.85 (1.98-6.46)* | 4.56 (2.04-10.18)* |
| Model 3 | Model 3 | | | |
| Elevated WC | Reference | 6.13 (3.94-9.53)* | 17.86 (6.96-45.83)* | 45.92 (6.16-342.40)* |
| Elevated BP | Reference | 1.51 (1.03-2.21)* | 2.39 (1.34-4.26)* | 1.79 (0.81-3.93) |
| Elevated glucose | Reference | 0.74 (0.08-6.46) | 1.59 (0.17-14.59) | 6.57 (1.10-39.03)* |
| Elevated TG | Reference | 1.40 (0.95-2.04) | 1.68 (0.96-2.93) | 2.08 (0.95-4.57) |
| Elevated HDL-C | Reference | 1.37 (0.91-2.06) | 1.22 (0.66-2.23) | 2.15 (0.97-4.77) |
| MetS | Reference | 2.80 (1.82-4.30)* | 3.74 (2.05-6.80)* | 4.80 (2.14-10.74)* |
| Girls and women | | | | |
|  | Model 1 | | | |
|  | Overweight | Class I obesity | Class II obesity | Class III obesity |
|  | (*n*=410) | (*n*=118) | (*n*=61) | (*n*=44) |
| Elevated WC | Reference | 3.77 (2.44-5.82)* | 5.49 (2.96-10.17)* | 76.93 (10.49-564.39)* |
| Elevated BP | Reference | 1.23 (0.80-1.89) | 1.96 (1.13-3.38)* | 3.66 (1.93-6.96)* |
| Elevated glucose | Reference | 5.44 (1.51-19.60)* | 5.25 (1.15-24.05)* | 4.83 (0.86-27.18) |
| Elevated TG | Reference | 1.36 (0.90-2.07) | 1.16 (0.67-2.02) | 2.35 (1.25-4.42)* |
| Elevated HDL-C | Reference | 1.33 (0.81-2.18) | 1.33 (0.70-2.54) | 4.89 (2.57-9.30)* |
| MetS | Reference | 2.18 (1.27-3.74)* | 2.88 (1.51-5.52)* | 9.73 (4.98-19.01)* |
|  | Model 2 | | | |
| Elevated WC | Reference | 3.82 (2.46-5.94)* | 5.58 (2.98-10.43)* | 69.08 (9.40-507.95)* |
| Elevated BP | Reference | 1.23 (0.80-1.90) | 1.96 (1.13-3.38)* | 3.68 (1.93-7.03)* |
| Elevated glucose | Reference | 5.38 (1.49-19.40)* | 5.12 (1.11-23.52)* | 4.40 (0.77-25.09) |
| Elevated TG | Reference | 1.38 (0.91-2.10) | 1.19 (0.68-2.07) | 2.62 (1.39-4.97)* |
| Elevated HDL-C | Reference | 1.32 (0.80-2.17) | 1.33 (0.69-2.53) | 4.81 (2.51-9.20)* |
| MetS | Reference | 2.17 (1.26-3.72)* | 2.85 (1.49-5.46)* | 9.17 (4.67-18.00)* |
|  | Model 3 | | | |
| Elevated WC | Reference | 4.01 (2.56-6.28)* | 5.71 (3.03-10.75)* | 74.22 (10.05-547.99)* |
| Elevated BP | Reference | 1.21 (0.78-1.87) | 2.03 (1.17-3.52)* | 3.81 (1.97-7.36)* |
| Elevated glucose | Reference | 5.75 (1.57-21.11)* | 5.09 (1.09-23.86)* | 4.44 (0.75-26.31) |
| Elevated TG | Reference | 1.39 (0.91-2.12) | 1.18 (0.68-2.06) | 2.60 (1.37-4.93)* |
| Elevated HDL-C | Reference | 1.36 (0.82-2.25) | 1.34 (0.69-2.58) | 4.52 (2.33-8.75)* |
| MetS | Reference | 2.23 (1.29-3.85)* | 2.92 (1.51-5.62)* | 8.68 (4.39-17.19)* |

WC, waist circumference; BP, blood pressure; TG, triglyceride; HDL-C, high-density lipoprotein cholesterol; MetS, metabolic syndrome.

Class I obesity was defined as ≥95th percentile and <120% of 95th percentile TMI.

Class II obesity was defined as ≥120% of 95th percentile and <140% of 95th percentile TMI.

Class III obesity was defined as ≥140% of 95th percentile of TMI.

Model 1: Statistical significance was determined using logistic regression analysis after no adjustment according to obesity class. * P<0.05

Model 2: Statistical significance was determined using multiple logistic regression analysis after adjustment for age and sex according to obesity class. * P<0.05

Model 3: Statistical significance was determined using multiple logistic regression analysis after adjustment for age, sex, alcohol drinking, smoking, household income, rural residence, physical activity, and diagnosis of type 2 diabetes mellitus (T2DM), hypertension, and dyslipidemia according to obesity class. * P<0.05
